# Single and repeated high-level blast, low-level blast, and new-onset self-reported health conditions in the U.S. Millennium Cohort Study: An exploratory investigation

**DOI:** 10.3389/fneur.2023.1110717

**Published:** 2023-03-21

**Authors:** Jennifer N. Belding, Claire A. Kolaja, Rudolph P. Rull, Daniel W. Trone

**Affiliations:** ^1^Leidos, San Diego, CA, United States; ^2^Deployment Health Research Department, Naval Health Research Center, San Diego, CA, United States

**Keywords:** blast, overpressure, high-level blast, low-level blast, diagnosis, military, occupational health

## Abstract

**Introduction:**

Although previous research suggests that overpressure exposure from either high-level blast (HLB) or low-level blast (LLB) are harmful to health, to date no large-scale studies with representative samples of military personnel have utilized prospective designs and self-reported measures to examine the relationships between blast exposure and health conditions. To address these limitations, this analysis of data from the Millennium Cohort Study (MCS), the largest and longest running study of U.S. service members and veterans, examined (1) whether single or repeated HLB exposure is associated with self-reported diagnoses of illness and injury, (2) whether repeated HLB is associated with greater risk than single HLB, (3) potential adverse consequences of LLB exposure using military occupation as a proxy, and (4) the combined effects of single or repeated HLB and LLB exposure.

**Method:**

MCS participants who completed the 2011–2013 survey (*N* = 138,949) were classified as having been exposed to “no,” “single,” or “repeated” HLB exposure, and into low or high risk of exposure to LLB based on occupation. Participants self-reported diagnosis of 45 medical conditions; newly reported diagnoses were regressed on single and repeated (vs. no) HLB, occupational risk of LLB, and relevant interactions using logistic regression.

**Results:**

Single and repeated HLB were associated with new onset of 25 and 29 diagnoses, respectively; repeated HLB exposure was associated with greater risk than single HLB exposure for five diagnoses (e.g., PTSD, depression). Occupational risk of LLB was associated with 11 diagnoses (e.g., PTSD, significant hearing loss). Additionally, 14 significant interactions were detected across 11 diagnoses.

**Discussion:**

Findings suggest that overpressure exposure (including single HLB, repeated HLB, and occupational risk of LLB) may increase the risks of self-reporting clinical diagnoses of PTSD, hearing loss, chronic fatigue syndrome, neuropathy-caused reduced sensation in the hands and feet, depression, vision loss, sinusitis, reflux, and anemia. Furthermore, the combination of HLB and LLB exposure may be associated with greater risk of migraines, PTSD, and impaired fecundity. These findings provide further evidence of the potential adverse consequences associated with overpressure exposure and underscore the necessity of public health surveillance initiatives for blast exposure and/or safety recommendations for training and operational environments.

## 1. Introduction

Members of the U.S. Armed Forces may be exposed to blast, also known as overpressure, as part of their routine occupational duties (1–3). Blast overpressure exposure can come from either incoming munitions (e.g., improvised explosive devices) or outgoing munitions (e.g., Carl Gustaf recoilless rifle) (4). Research to date suggests that both forms of blast overpressure exposure are harmful to health (1, 2, 5).

High-level blast (HLB) refers to overpressure generated by incoming munitions and was identified as the leading cause of deployment-related injuries, morbidity, and mortality during the Global War on Terror (4, 6, 7). A greater number of service members returned from combat deployments with mild to severe HLB-induced injuries that were ultimately not fatal due to advances in personal protective equipment and combat casualty care (8). Furthermore, as treatment of HLB-related injuries improved over time, more warfighters were able to return to battle where subsequent exposures to repeated HLB may occur. Although previous research has suggested an association between HLB exposure and a range of injuries including traumatic amputation, traumatic brain injury, organ damage, lung injury, auditory injury, and adverse mental health conditions (2, 9, 10), more research is needed to understand the consequences of repetitive HLB exposure.

Low-level blast (LLB), on the other hand, refers to overpressure generated by outgoing munitions (4). Although exposure to overpressure generated by such munitions is generally lower in intensity than HLB, recent literature reviews suggest that LLB may be associated with adverse health outcomes such as subclinical neurological and auditory symptomology, particularly among service members who experience cumulative exposures due to occupational duties (e.g., instructors leading training courses) (1, 11, 12). While much of this previous research was based on small-scale studies of various training programs, epidemiological investigations suggest military occupations with repetitive LLB exposure (e.g., from firing specific weapons systems known to generate overpressure) have elevated risks of traumatic brain injury diagnoses, particularly concussion and moderate TBI, tinnitus, and other neurological and auditory symptoms (13–17).

This emerging body of research has notable limitations. Previous studies predominantly employed retrospective study designs, rarely examined the effects of both HLB and LLB simultaneously within the same statistical model, or relied on archival medical and career records (13–17). No large-scale efforts to date utilized prospective designs and self-reported measures of exposures and/or outcomes. Additionally, nearly all human studies focused exclusively on active duty populations or law enforcement personnel, which are not necessarily representative of the broader military community (1, 18). For example, Reservists and National Guard personnel regularly deploy and may be exposed to blast in training and operational environments, yet were rarely included in research to date. As the effects of LLB are hypothesized to be cumulative in nature with latent outcomes emerging with age (1, 11, 12, 19, 20), there is a need to examine the effects of overpressure exposure even after leaving military service. Furthermore, limited research to date has directly examined the distinction between single and repetitive HLB, despite earlier work suggesting that outcomes may be more severe for those with repetitive HLB exposure (21–23).

In order to address the limitations of prior work, the present research examined Millennium Cohort Study data to estimate the effects of HLB and LLB exposure, both independently and jointly, on a variety of self-reported new-onset diagnoses of illness and injury. The purpose of this exploratory research was to examine whether HLB exposure (either single or repeated) is associated with self-reported diagnoses of illness and injury, examine whether repeated HLB is associated with greater risk than single HLB, and examine the potential adverse consequences of LLB exposure using MOS as a proxy for LLB. We hypothesized that both HLB and LLB exposures would be associated with elevated risks of neurological, hearing-related, and mental health diagnoses. We further speculated that the effects of HLB may be heterogeneous across occupational risk levels of LLB. Drawing on previous research in animals and humans, we primarily expected these interactions with regard to post-traumatic stress disorder (PTSD) and migraines, respectively (1, 11–13).

## 2. Materials and methods

The Millennium Cohort Study is the largest and longest-running prospective health study of members of the U.S. Armed Forces (24, 25) and was designed to investigate the long-term effects of military service on service member and veteran health (25). Thorough descriptions of the Study are available elsewhere (26, 27). Briefly, the Study uses a multi-panel multi-wave design with participants representing all branches of service, components, paygrades, and occupations. After providing informed consent, participants are requested to complete a self-administered survey every 3–5 years, even after separation from service, and responses can be linked with a variety of other data sources.

### 2.1. Study population

**Figure 1:**
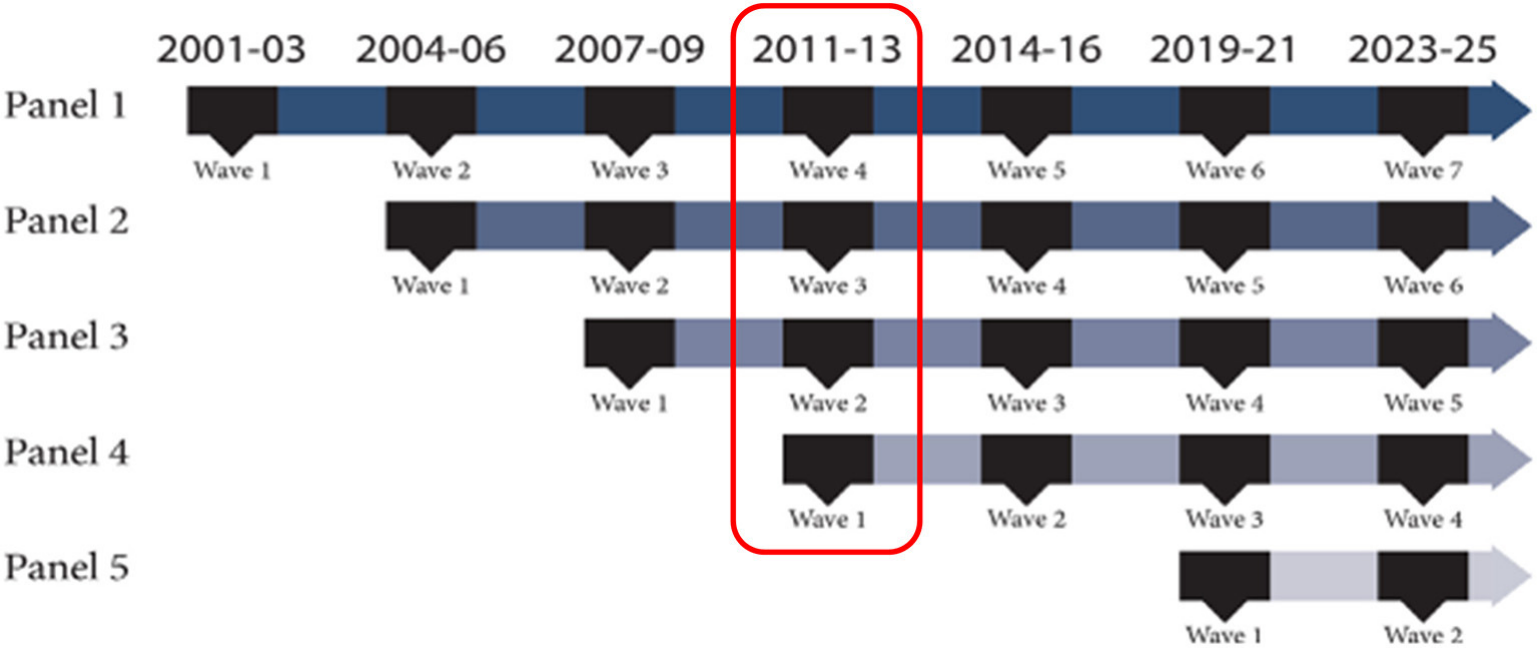
Depiction of the Millennium cohort study's multi-panel, multi-wave design. The red box identifies data utilized for the present research.

The present investigation utilized data from participants enrolled in Panels 1–4 (recruited in 2001, 2004, 2007, and 2011, respectively) who completed the 2011–2013 survey data collection (henceforth referred to as 2013 survey) (*N* = 138,949; see Figure 1).

### 2.2. Measures

#### 2.2.1. High-level blast

Exposure to HLB was first assessed in the 2013 survey. Specifically, the survey asked whether participants were ever injured from training or sports injuries, blast/explosion/bullet, and motor vehicle accident/crash. Participants indicated whether an injury occurred, occurred while deployed, and/or occurred while not deployed. If participants indicated that they were injured, they were then asked to report the total number of injury events using a two-digit textbox. Participants who indicated that they were injured from a blast/explosion/bullet while deployed and/or not deployed were categorized as having been exposed to HLB and were further categorized into groups reflecting either single or repeated HLB using the self-reported number of injury events.

#### 2.2.2. Low-level blast

Consistent with previous research (14–17), occupational risk for LLB was determined using self-reported military occupation as a proxy. Millennium Cohort surveys assessed occupation at the time of survey completion; response options for enlisted personnel and officers or warrant officers were consistent with official DoD military occupational coding schemes, while response options for civilians were consistent with categories provided by the Bureau of Labor Statistics. Participants were categorized into high vs. low risk of repetitive LLB if they reported ever having worked in occupations determined to be high risk on any survey administered between 2001 and 2013. Enlisted high-risk occupations included infantry; armor or amphibious; combat engineering; artillery/gunnery, rockets, or missiles; air crew; seamanship; installation security; medical care; ancillary medical support; and law enforcement. Officer or Warrant Officer high-risk occupations included fixed wing fighter or bomber pilot, helicopter pilot, and aircraft crew. All remaining occupational categories, including all civilian categories, were considered low-risk occupations.

#### 2.2.3. Self-reported diagnoses

The Millennium Cohort survey asked participants to indicate whether they had ever been told (at baseline) or in the past 3 years (at follow-up) by a doctor or other health professional that they have had any of 45 different self-reported conditions. Although we examined all available conditions for exploratory purposes, we hypothesized *a priori* that 11 conditions would be most likely to show associations with blast exposure (chronic fatigue syndrome, depression, significant hearing loss, manic-depressive disorder, migraine headaches, neuropathy-caused reduced sensation in hands or feet, PTSD, schizophrenia/psychosis, stroke, seizures, tinnitus/ringing of the ears).

### 2.3. Statistical analysis

After calculating descriptive statistics among all 2013 survey responders, the likelihood of newly self-reported physician diagnosis was regressed on frequency of self-reported HLB exposure, occupational risk of LLB, and their interaction using separate logistic regressions for each condition. Sample sizes for each analysis differed because those who reported the diagnosis on surveys administered before 2013 were excluded. Newly-reported kidney failure requiring dialysis was excluded due to a relatively small number of cases. For these analyses, single and repeated HLB were dummy coded and entered separately with no HLB exposure as the referent. Additionally, occupational risk of LLB was dummy coded with low occupational risk of LLB exposure as the referent. Finally, to assess potential effect measure modification, interaction terms between single and repeated HLB and LLB, respectively, were included in models for each health condition. When significant interactions emerged, analyses were repeated stratified by occupational risk of LLB. Additionally, logistic regression analyses were repeated where different combinations of HLB and LLB were compared against a consistent referent of no HLB exposure and low LLB risk. Analyses adjusted for sex, birth year, race, ethnicity, component, paygrade, branch of service, deployment with and without combat experience, and panel. A threshold of *p* ≤ 0.05 was used to determine statistical significance; analyses were conducted using IBM SPSS Software Version 25.

## 3. Results

Overall, the sample was predominantly born after 1980, male, non-Hispanic White, married, served in the Army, part of the active duty component and enlisted pay grade (Table 1). Half had at least an Associate's degree as of the 2013 survey. Approximately 40% did not deploy, 12% deployed without combat, and 48% deployed with combat prior to the 2013 survey. Furthermore, 41% reported ever being in an occupation with high LLB exposure prior to or during the completion of the 2013 survey, while 3% reported single HLB and 3% reported repeated HLB exposure. The analytic sample sizes for each condition differed because of the restriction to newly-reported diagnosis (Table 2), but these overall descriptive distributions remained in the subsamples.

**Table 1:** Sample characteristics.

	***N***	**%**
**Birth year** ^1^		
Before 1960	13,651	9.8
1960–1969	23,195	16.7
1970–1979	30,842	22.2
1980+	71,261	51.3
**Sex** ^1^		
Male	97,651	70.3
Female	41,298	29.7
**Race and ethnicity** ^1^		
American Indian	1,890	1.4
Asian or Pacific Islander	6,115	4.4
Black, non-Hispanic	15,076	10.9
Hispanic	10,566	7.6
Other	1,682	1.2
White, non-Hispanic	103,559	74.6
**Educational attainment** ^2^		
Less than high school completion/diploma	148	0.1
High school degree/GED/or equivalent	16,326	11.8
Some college, no degree	50,173	36.1
Associate's degree	19,327	13.9
Bachelor's degree	31,294	22.5
Master's, doctorate, or professional degree	21,676	15.6
**Marital status** ^2^		
Single, never married	29,558	21.3
Married	89,221	64.2
Separated	4,337	3.1
Divorced	15,257	11.0
Widowed	576	0.4
**Military rank** ^1^		
Enlisted	110,908	79.8
Warrant Officer	1,129	0.8
Officer	26,912	19.4
**Service branch** ^1^		
Army	61,869	44.5
Navy	22,197	16.0
Marine Corps	11,633	8.4
Air Force	40,634	29.2
Coast Guard	2,616	1.9
**Component** ^1^		
Active duty	90,392	65.1
Reserve/Guard	48,557	34.9
Not deployed	55,195	39.7
Deployed without combat	17,033	12.3
Deployed with combat	66,721	48.0
**Occupational risk of LLB**		
Low	79,311	57.1
High	55,768	41.3
**High-level blast exposure**		
No HLB exposure	122,175	93.7
Single HLB exposure	4,047	3.1
Repeated HLB exposure	4,141	3.2

^1^Identified upon enrollment in the Millennium Cohort Study.

^2^Identified during the 2011–2013 survey data collection.

**Table 2:** Effect estimates for levels and 2-way interactions of HLB and LLB exposures.

			**Single HLB**			**Repeated HLB**			**LLB**			**Single HLB x LLB Interaction**			**Repeated HLB x LLB Interaction**		
	***N***	**New Onset**	**AOR**	**95% CI**	***p***	**AOR**	**CI**	***p***	**AOR**	**CI**	***p***	**AOR**	**CI**	***p***	**AOR**	**CI**	***p***
**Theoretically relevant**																	
Chronic fatigue syndrome	126,688	1,256	3.31	2.28, 4.81	*	6.14	4.36, 8.65	*	1.24	1.08, 1.43	0.002	0.57	0.35, 0.94	0.03	0.77	0.51, 1.15	0.19
Depression	117,259	13,529	2.36	2.04, 2.73	*	3.65	3.12, 4.26	*	1.11	1.07, 1.16	*	0.97	0.81, 1.16	0.72	0.94	0.78, 1.12	0.48
Significant hearing loss	118,841	6,875	2.86	2.40, 3.42	*	4.00	3.32, 4.82	*	1.34	1.27, 1.43	*	0.92	0.74, 1.14	0.43	1.03	0.83, 1.27	0.82
Manic-depressive disorder	126,858	1,210	2.39	1.57, 3.63	*	3.76	2.50, 5.65	*	1.09	0.95, 1.26	0.21	0.92	0.55, 1.55	0.76	0.97	0.61, 1.55	0.88
Migraine headaches	116,504	10,987	2.62	2.24, 3.08	*	3.58	3.01, 4.26	*	1.01	0.96, 1.06	0.69	1.15	0.95, 1.40	0.15	1.58	1.30, 1.92	*
Neuropathy-caused reduced sensation in hands or feet	125,399	2,989	3.08	2.40, 3.95	*	4.65	3.62, 5.97	*	1.19	1.09, 1.30	*	0.93	0.68, 1.26	0.64	0.98	0.74, 1.31	0.90
Post-traumatic stress disorder	123,540	9,564	4.85	4.21, 5.58	*	8.55	7.36, 9.93	*	1.45	1.37, 1.53	*	0.83	0.70, 0.98	0.03	0.83	0.70, 0.99	0.03
Schizophrenia or psychosis	127,429	373	3.83	2.05, 7.17	*	4.99	2.58, 9.68	*	1.07	0.82, 1.39	0.61	0.78	0.35, 1.72	0.53	0.93	0.44, 1.98	0.85
Stroke	127,431	311	2.60	1.26, 5.35	0.01	1.96	0.72, 5.36	0.19	0.82	0.63, 1.07	0.14	0.35	0.10, 1.19	0.09	1.47	0.47, 4.64	0.51
Seizures	127,446	673	2.91	1.77, 4.79	*	3.64	2.13, 6.21	*	0.99	0.82, 1.19	0.88	1.02	0.55, 1.89	0.94	0.93	0.50, 1.72	0.81
Tinnitus/ringing of the ears	128,356	19,524	3.76	3.34, 4.23	*	4.58	4.01, 5.24	*	1.20	1.16, 1.24	*	0.92	0.80, 1.24	0.26	1.14	0.98, 1.33	0.10
**Theoretically irrelevant**																	
Acid reflux/gastroesophageal reflux disease requiring medication	128,304	17,146	1.79	1.55, 2.05	*	1.89	1.61, 2.22	*	1.06	1.02, 1.10	0.002	0.91	0.77, 1.09	0.30	1.02	0.85, 1.23	0.80
Anemia	122,882	3,563	2.03	1.51, 2.74	*	1.57	1.04, 2.36	0.03	1.10	1.03, 1.20	0.009	0.57	0.36, 0.90	0.02	0.76	0.45, 1.29	0.31
Angina (chest pain)	124,422	2,307	1.84	1.34, 2.53	*	2.37	1.70, 3.31	*	0.93	0.84, 1.03	0.15	0.96	0.65, 1.43	0.84	0.99	0.68, 1.43	0.97
Any other heart condition (please specify)	121,604	2,961	1.18	0.84, 1.68	0.34	2.06	1.50, 2.83	*	1.07	0.99, 1.16	0.11	1.31	0.87, 1.99	0.20	0.64	0.43, 0.94	0.02
Any other hepatitis	126,880	308	0.63	0.16, 2.57	0.52	0.45	0.06, 3.23	0.43	0.82	0.63, 1.06	0.13	1.61	0.31, 8.48	0.57	3.96	0.50, 31.49	0.19
Asthma	122,602	4,142	1.44	1.11, 1.88	0.007	1.56	1.17, 2.10	0.003	0.95	0.88, 1.02	0.16	0.95	0.68, 1.33	0.78	0.93	0.66, 1.32	0.70
Bladder infection	120,503	3,993	1.31	0.94, 1.83	0.12	1.62	1.12, 2.34	0.01	1.11	1.03, 1.19	0.007	0.91	0.58, 1.43	0.68	1.23	0.79, 1.91	0.36
Cancer (please specify)	124,976	1,738	1.35	0.87, 2.10	0.18	1.57	0.96, 2.57	0.07	1.01	0.91, 1.13	0.80	1.18	0.68, 2.04	0.56	1.03	0.58, 1.85	0.91
Chronic bronchitis	124,742	2,299	1.99	1.46, 2.70	*	1.97	1.37, 2.83	*	0.96	0.87, 1.06	0.41	0.82	0.55, 1.22	0.32	1.18	0.78, 1.79	0.43
Cirrhosis	127,560	170	0.62	0.09, 4.45	0.63	2.49	0.77, 8.05	0.13	0.89	0.62, 1.29	0.54	3.99	0.48, 32.96	0.20	0.81	0.20, 3.21	0.76
Coronary heart disease	127,145	614	1.61	0.85, 3.05	0.15	1.83	0.89, 3.75	0.10	0.96	0.80, 1.14	0.62	0.54	0.22, 1.33	0.18	0.91	0.39, 2.13	0.82
Crohn's disease	127,823	223	1.32	0.42, 4.19	0.64	2.40	0.87, 6.62	0.09	1.07	0.80, 1.45	0.65	0.45	0.09, 2.28	0.33	0.67	0.20, 2.23	0.52
Diabetes or sugar diabetes	126,359	1,905	1.34	0.88, 2.03	0.17	1.03	0.59, 1.81	0.91	0.98	0.88, 1.09	0.69	0.75	0.43, 1.30	0.30	1.61	0.86, 2.99	0.14
Emphysema	127,472	299	2.63	1.27, 5.43	0.009	0.92	0.23, 3.76	0.91	0.79	0.60, 1.05	0.10	0.99	0.39, 2.51	0.99	2.49	0.55, 11.38	0.24
Fibromyalgia	127,428	943	2.61	1.63, 4.18	*	2.83	1.61, 5.00	*	0.95	0.82, 1.11	0.97	0.99	0.52, 1.88	0.97	1.36	0.70, 2.65	0.36
Gallstones	126,007	1,596	1.02	0.60, 1.74	0.94	2.14	1.34, 3.41	0.001	1.01	0.90, 1.13	0.86	1.79	0.96, 3.36	0.07	0.67	0.37, 1.19	0.17
Heart attack	127,086	423	1.71	0.80, 3.68	0.17	1.42	0.52, 3.87	0.49	1.00	0.80, 1.25	>0.99	0.53	0.19, 1.52	0.24	1.54	0.51, 4.62	0.44
Hepatitis B	127,182	251	1.20	0.38, 3.82	0.75	0.56	0.08, 4.01	0.56	1.02	0.77, 1.35	0.90	0.53	0.10, 2.69	0.44	2.90	0.36, 23.40	0.32
Hepatitis C	127,240	192	0.52	0.07, 3.72	0.51	1.40	0.34, 5.75	0.64	1.02	0.74, 1.42	0.89	3.21	0.38, 26.78	0.28	1.12	0.23, 5.37	0.89
High cholesterol requiring medication	128,417	15,159	1.31	1.10, 1.56	0.003	1.92	1.59, 2.33	*	1.00	0.96, 1.05	0.92	0.90	0.72, 1.12	0.34	0.81	0.65, 1.02	0.07
Hypertension	116,268	10,128	1.41	1.17, 1.68	*	1.55	1.26, 1.90	*	1.02	0.98, 1.07	0.34	1.02	0.82, 1.27	0.86	1.23	0.98, 1.55	0.08
Kidney failure requiring dialysis	127,651	113															
Kidney stones	128,168	4,908	1.94	1.55, 2.42	*	1.50	1.12, 2.02	0.007	1.04	0.97, 1.10	0.26	0.75	0.56, 0.99	0.04	1.20	0.86, 1.66	0.29
Impaired fecundity	128,179	2,113	1.13	0.73, 1.73	0.59	1.17	0.70, 1.93	0.55	1.04	0.94, 1.15	0.44	1.67	1.01, 2.82	0.05	1.91	1.09, 3.34	0.02
Lupus	127,706	210	2.50	1.01, 6.20	0.05	0.72	0.10, 5.18	0.74	0.75	0.54, 1.04	0.09	0.70	0.18, 2.71	0.60	4.15	0.50, 34.18	0.19
Multiple sclerosis	127,908	223	1.91	0.70, 5.25	0.21	2.05	0.64, 6.55	0.23	1.00	0.74, 1.36	0.98	0.45	0.10, 2.10	0.31	0.74	0.18, 3.04	0.67
Pancreatitis	127,260	368	0.32	0.05, 2.29	0.32	1.37	0.43, 4.32	0.60	1.11	0.88, 1.40	0.40	6.65	0.86, 51.34	0.07	1.04	0.28, 3.83	0.95
Rheumatoid arthritis	125,466	1,807	2.57	1.87, 3.53	*	3.91	2.83, 5.39	*	0.95	0.85, 1.06	0.37	0.89	0.59, 1.34	0.57	0.74	0.50, 1.09	0.13
Significant vision loss even with glasses or contact lenses	125,483	3,425	1.96	1.50, 2.57	*	3.13	2.40, 4.09	*	1.12	1.04, 1.22	0.004	0.96	0.69, 1.33	0.79	0.88	0.64, 1.19	0.40
Sinusitis	113,139	7,132	1.94	1.59, 2.36	*	1.89	1.50, 2.38	*	1.10	1.04, 1.16	0.001	0.66	0.50, 0.85	0.002	0.95	0.72, 1.25	0.70
Sleep apnea	123,376	7,779	2.08	1.74, 2.49	*	2.32	1.90, 2.84	*	1.01	0.96, 1.07	0.75	0.80	0.64, 1.00	0.05	1.29	1.03, 1.63	0.03
Stomach, duodenal, or peptic ulcer	124,728	1,804	1.46	0.96, 2.20	0.08	2.74	1.90, 3.95	*	1.09	0.97, 1.21	0.14	1.24	0.76, 2.03	0.39	0.94	0.62, 1.43	0.77
Thyroid condition other than cancer	124,998	2,561	0.81	0.50, 1.30	0.37	2.01	1.37, 2.95	*	1.03	0.94, 1.12	0.54	1.88	1.08, 3.29	0.03	1.05	0.67, 1.65	0.82
Ulcerative colitis or proctitis	127,099	494	0.95	0.35, 2.56	0.91	4.86	2.79, 8.48	*	1.09	0.89, 1.34	0.41	1.28	0.39, 4.15	0.69	0.50	0.25, 1.00	0.05

^*^p ≤ 0.001.

Findings are presented in order of the associations tested within each model. The five largest magnitudes of association are highlighted in the text and are occasionally supplemented with additional significant findings that were a priori hypothesized to be associated with blast exposure (see Table 2 for all adjusted associations). At least one significant association emerged for 34 of the 45 diagnoses examined, leaving 11 with no significant associations for single or repeated HLB, LLB, and their respective interactions. These 11 diagnoses included coronary heart disease, heart attack, pancreatitis, diabetes, hepatitis B, hepatitis C, any other hepatitis, cirrhosis, multiple sclerosis, Crohn's disease, and cancer.

### 3.1. Results for the effects of single and repeated HLB

Single HLB exposure was significantly associated with 25 of 45 self-reported diagnoses including all 11 of the conditions hypothesized *a priori* to be affected by blast (Table 2). The highest magnitudes of association for single vs. no HLB were observed for PTSD (adjusted odds ratio [AOR] = 4.85), schizophrenia or psychosis (3.83), tinnitus (3.76), chronic fatigue syndrome (3.31), and neuropathy-caused reduced sensation in the hands and feet (3.08). Repeated (vs. no) HLB exposure was significantly associated with 29 of 45 self-reported diagnoses and 10 of the conditions hypothesized *a priori* to be affected by blast. AORs for repeated HLB vs. no HLB were highest for PTSD (8.55), chronic fatigue syndrome (6.14), schizophrenia or psychosis (4.99), ulcerative colitis or proctitis (4.86), neuropathy-caused reduced sensation in the hands or feet (4.65), tinnitus (4.58), and significant hearing loss (4.00).

Of particular note, there were five significant differences between single and repeated HLB exposure (see Table 2). Repeated HLB exposure was associated with significantly greater risk than single HLB exposure for PTSD (AOR_single_ = 4.85, AOR_repeated_ = 8.55), depression (AOR_single_ = 2.36, AOR_repeated_ = 3.65), and high cholesterol requiring medication (AOR_single_ = 1.31, AOR_repeated_ = 1.92). Additionally, whereas repeated blast exposure was significantly associated with ulcerative colitis and thyroid conditions other than cancer (AORs_repeated_ = 4.86 and 2.01, respectively), single HLB exposure was not.

### 3.2. Results for the effect of LLB

Occupational risk of LLB exposures was significantly associated with 11 of the 45 diagnoses examined, including 6 of the 11 conditions hypothesized *a priori* to be affected by blast. The highest magnitudes of association were observed for PTSD (1.45), significant hearing loss (1.34), chronic fatigue syndrome (1.24), tinnitus (1.20), neuropathy-caused reduced sensation in the hands and feet (1.19), significant vision loss (1.12), and depression (1.11).

### 3.3. Results for the interaction between HLB and LLB

There were 14 significant interactions detected, including 8 single HLB by LLB interactions and 6 repeated HLB by LLB interactions across 11 diagnoses. Decomposition of the joint effect estimates are depicted in Table 3, which presents the AORs of single HLB vs. no HLB and repeated HLB vs. no HLB stratified by occupational risk for LLB. Table 4 presents the AORs of the combinations of blast exposure compared against a consistent referent of no HLB exposure and low LLB risk.

**Table 3:** Decomposition of significant interactions.

	**Single HLB vs. No HLB**			**Repeated HLB vs. No HLB**		
	**OR**	**CI**	***p***	**OR**	**95% CI**	***p***
**Chronic fatigue syndrome**						
Low occupational risk of LLB	3.38	2.31, 4.92	*	–	–	–
High occupational risk of LLB	1.84	1.30, 2.60	0.001	–	–	–
**Sinusitis**						
Low occupational risk of LLB	1.91	1.57, 2.34	*	–	–	–
High occupational risk of LLB	1.28	1.07, 1.53	0.007	–	–	–
**Anemia**						
Low occupational risk of LLB	2.01	1.49, 2.71	*	–	–	–
High occupational risk of LLB	1.20	0.84, 1.70	0.32	–	–	–
**Thyroid condition**						
Low occupational risk of LLB	0.81	0.51, 1.30	0.39	–	–	–
High occupational risk of LLB	1.51	1.11, 2.06	0.008	–	–	–
**Kidney stones**						
Low occupational risk of LLB	1.90	1.52, 2.37	*	–	–	–
High occupational risk of LLB	1.48	1.23, 1.78	*	–	–	–
**Migraines**						
Low occupational risk of LLB	–	–	–	3.62	3.03, 4.31	*
High occupational risk of LLB	–	–	–	5.49	4.97, 6.07	*
**Any other heart conditions**						
Low occupational risk of LLB	–	–	–	2.06	1.94, 2.83	*
High occupational risk of LLB	–	–	–	1.29	1.02, 1.64	0.04
**Ulcerative colitis**						
Low occupational risk of LLB	–	–	–	4.84	2.75, 8.53	*
High occupational risk of LLB	–	–	–	2.46	1.53, 3.94	*
**Sleep apnea**						
Low occupational risk of LLB	2.09	1.74, 2.50	*	2.32	1.89, 2.85	*
High occupational risk of LLB	1.63	1.42, 1.88	*	2.93	2.62, 3.28	*
**Impaired fecundity**						
Low occupational risk of LLB	1.14	0.74, 1.76	0.54	1.18	0.71, 1.95	0.53
High occupational risk of LLB	1.86	1.38, 2.49	*	2.17	1.66, 2.83	*
**PTSD**						
Low occupational risk of LLB	4.91	4.26, 5.66	*	8.54	7.35, 9.93	*
High occupational risk of LLB	3.92	3.54, 4.34	*	6.93	6.31, 7.61	*

^*^p < 0.001.

– Analyses were not conducted while stratifying by occupational risk of LLB because the lack of a significant interaction in the omnibus analyses indicates that decomposition analyses were unnecessary as there are no significant differences across the groups.

**Table 4:** AORs for levels of HLB and LLB exposure when compared against a consistent referent of no HLB and low LLB risk.

	**No HLB, low LLB risk**	**Single HLB, low LLB risk**			**Repeated HLB, low LLB risk**			**No HLB, high LLB risk**			**Single HLB, high LLB risk**			**Repeated HLB, high LLB risk**		
	**AOR**	**AOR**	**CI**	***p***	**AOR**	**CI**	***p***	**AOR**	**CI**	***p***	**AOR**	**CI**	***p***	**AOR**	**CI**	***p***
Chronic fatigue syndrome	1.00 (Ref)	2.55	1.76, 3.69	*	4.67	3.33, 6.54	*	1.00	0.88, 1.14	0.98	1.74	1.24, 2.44	0.001	4.29	3.43, 5.37	*
Sinusitis	1.00 (Ref)	1.83	1.50, 2.23	*	1.77	1.41, 2.24	*	1.05	1.00, 1.11	0.06	1.31	1.10, 1.56	0.003	1.84	1.59, 2.13	*
Anemia	1.00 (Ref)	1.96	1.45, 2.63	*	1.50	1.00, 2.27	0.051	1.08	1.00, 1.16	0.054	1.23	0.87, 1.73	0.24	1.25	0.89, 1.75	0.19
Thyroid condition	1.00 (Ref)	0.78	0.49, 1.25	0.30	1.93	1.32, 2.83	0.001	1.00	0.92, 1.10	0.95	1.50	1.11, 2.02	0.008	2.08	1.62, 2.67	*
Kidney stones	1.00 (Ref)	1.86	1.49, 2.32	*	1.44	1.07, 1.93	0.02	1.01	0.94, 1.07	0.89	1.44	1.20, 1.72	*	1.78	1.52, 2.08	*
Migraines	1.00 (Ref)	2.32	1.98, 2.72	*	3.14	2.64, 3.74	*	0.92	0.87, 0.96	*	2.64	2.36, 2.96	*	4.90	4.46, 5.38	*
Any other heart condition	1.00 (Ref)	1.16	0.82, 1.64	0.40	2.01	1.47, 2.76	*	1.06	0.98, 1.15	0.18	1.62	1.29, 2.05	*	1.36	1.08, 1.72	0.009
Ulcerative colitis	1.00 (Ref)	0.88	0.33, 2.37	0.80	4.47	2.57, 7.76	*	1.03	0.84, 1.25	0.79	1.21	0.64, 2.30	0.56	2.41	1.54, 3.78	*
Sleep apnea	1.00 (Ref)	1.89	1.58, 2.26	*	2.09	1.71, 2.56	*	0.93	0.88, 0.98	0.008	1.50	1.31, 1.73	*	2.70	2.42, 3.01	*
Impaired fecundity	1.00 (Ref)	1.07	0.70, 1.64	0.76	1.10	0.67, 1.82	0.71	1.00	0.91, 1.10	0.97	1.86	1.40, 2.47	*	2.17	1.68, 2.79	*
PTSD	1.00 (Ref)	3.84	3.34, 4.42	*	6.71	5.79, 7.78	*	1.16	1.10, 1.22	*	4.46	4.03, 4.93	*	7.82	7.14, 8.56	*

^*^p < 0.001.

Significant interactions of single HLB and LLB were detected for anemia, chronic fatigue syndrome, kidney stones, sinusitis, thyroid condition, but no interactions for repeated HLB by LLB were detected for these conditions. Single (vs. no) HLB was associated with greater risk of anemia, chronic fatigue syndrome, kidney stones, and sinusitis among those in low risk occupations compared to high risk occupations. However, this pattern reversed for thyroid conditions such that single (vs. no) HLB was associated with greater risk of thyroid conditions among those in high vs. low risk occupations. However, interactions were not observed for repeated (vs. no) HLB.

Three diagnoses (any other heart conditions, migraine headaches, ulcerative colitis) had significant interactions for repeated HLB and LLB, but not single HLB and LLB. Repeated (vs. no) HLB was associated with greater risk of migraines among those in high risk compared to low risk occupations. However, any other heart conditions and ulcerative colitis showed the opposite pattern such that repeated (vs. no) HLB was associated with greater risk of these conditions among those in low vs. high risk occupations. Contrary to expectations, the joint AORs for repeated HLB and high LLB risk were not the largest AORs observed for any other heart conditions and ulcerative colitis (see Table 4).

**Figure 2:**
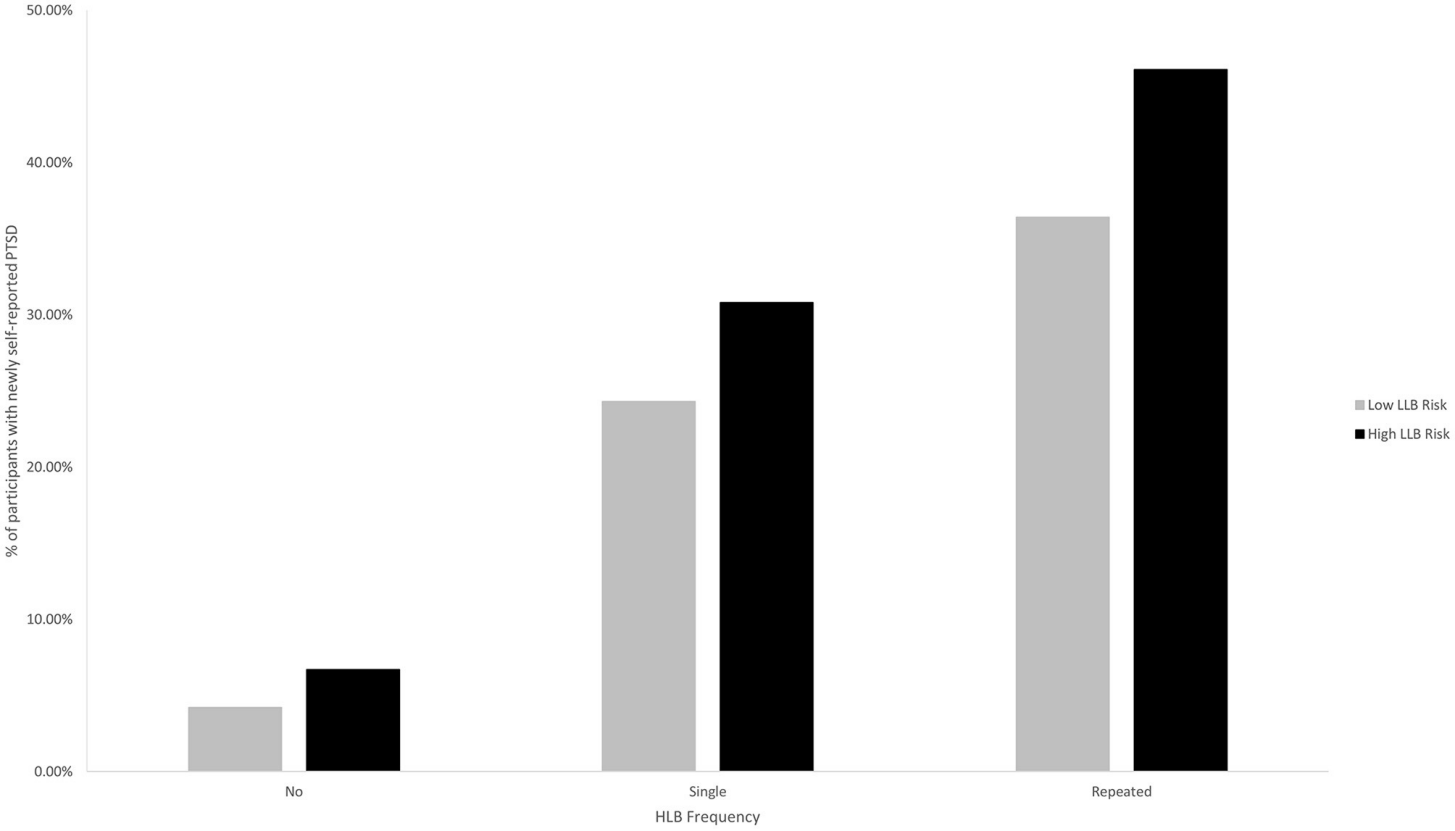
Percentage of participants with newly self-reported PTSD as a function of occupational risk of LLB and HLB frequency.

Finally, significant interactions were detected for three diagnoses (impaired fecundity, PTSD, sleep apnea) for single (vs. no) HLB and repeated (vs. no) HLB with LLB, respectively. First, both single and repeated (vs. no) HLB were associated with greater risk of impaired fecundity among those in high-exposure occupations but not among those in low-exposure occupations. Second, the magnitudes of association for both single and repeated (vs. no) HLB on PTSD were relatively greater among those working in low vs. high risk occupations. As expected, the AORs for no, single, and repeated HLB consistently increased across both low and high LLB risk occupations. Although the AOR for high LLB risk and repeated HLB exposure was the largest AOR observed, it appeared to be lower than expected (see Table 4). Close inspection of the data suggests that prevalence of PTSD was greater among those in high risk occupations in both the presence and absence of HLB. Specifically, among those working in low-risk occupations, 4.2% of those with no HLB exposure, 24.3% of those with single HLB exposure, and 36.4% of those with repeated HLB exposure self-reported PTSD. In contrast, these numbers rise to 6.7, 30.8, 46.1%, respectively, among those working in high-risk occupations (see Figure 2). Third, for sleep apnea the effect of single (vs. no) HLB was greater for those in low- compared with high-exposure occupations. However, this pattern reversed for repeated (vs. no) HLB such that risk for sleep apnea was greater among high- vs. low-exposure occupations.

## 4. Discussion

This exploratory analysis of data from 138,949 members of the Millennium Cohort, representing active duty, Reserve, and National Guard personnel, estimated associations between single HLB, repeated HLB, and occupational risk of LLB on newly-reported diagnoses. Findings from this analysis suggest that overpressure exposure (including single HLB, repeated HLB, and occupational risk of LLB) may increase the risks of self-reporting clinical diagnoses of PTSD, hearing loss, chronic fatigue syndrome, tinnitus, neuropathy-caused reduced sensation in the hands and feet, depression, vision loss, sinusitis, reflux, and anemia. Additionally, both single and repeated HLB, but not LLB, were associated with schizophrenia, migraines, seizure, rheumatoid arthritis, fibromyalgia, manic depressive disorder, chronic bronchitis, sleep apnea, angina, hypertension, high cholesterol requiring medication, asthma, and kidney stones. Furthermore, repeated HLB was associated with greater risk above and beyond that of single HLB of PTSD, depression, high cholesterol requiring medication, ulcerative colitis, and thyroid conditions.

We were uniquely able to examine the interaction between HLB and LLB and identified interactions for eleven conditions, though we draw special attention to four here: migraines, sleep apnea, impaired fecundity, and PTSD. First, repeated (vs. no) blast exposure was associated with significantly greater risk of migraines for those working in high risk occupations compared to their lower risk counterparts. Although this association was restricted to the comparison of repeated HLB and was not replicated for single HLB, this finding extends previous research on the association between blast exposure and headaches (14, 28–32). Second, although respective interactions of single and repeated HLB and LLB were significant for sleep apnea, that the direction of the association switches for single and repeated HLB warrants caution. Third, the data suggest that although single and repeated HLB were not associated with impaired fecundity among those working in low risk occupations, those working in occupations marked by LLB were significantly more likely to report impaired fecundity following single or repeated HLB exposure. Fourth, the effect of HLB on PTSD was more pronounced among those who worked in low risk occupations, which reflects the fact that those working in high risk occupations were more likely to report PTSD in both the presence and absence of HLB.

These findings contribute to a growing body of research linking overpressure exposure with adverse health and wellbeing outcomes. As with previous research, the most consistent findings emerged primarily for conditions that were neurological, hearing-related, or mental health-related. Specifically, these findings provide yet more evidence of the association between overpressure exposure (including single HLB, repeated HLB, and occupational LLB exposure) and hearing loss and tinnitus diagnosis (1, 11–13, 17, 33–35). Additionally, this research builds on previous research reporting on the adverse reproductive health consequences of HLB in that it reports that such effects occur for those who work occupations at high (but not low) risk for occupational exposure to LLB (36, 37).

Furthermore, the current research extends previous research on the association between LLB and subclinical headaches (14, 28–32). Although there was a significant association between single and repeated HLB and migraines, occupational risk of LLB was not significantly associated with newly diagnosed migraines. This lack of a statistically significant association was surprising, but this may be an artifact of a social expectation of headaches following certain training exercises involving exposure to LLB, which may reduce service members' healthcare seeking behavior. Nonetheless, the observed interaction of LLB and repeated HLB suggests that the association between repeated HLB and migraines was stronger for those working in high (vs. low) risk occupations, providing further evidence of the association between overpressure exposure and a more severe form of headaches (38, 39).

It is also clear from previous research that exposure to HLB, which is inherently a traumatic event, is associated with increased risk of PTSD (40–42). However, the work presented herein is one of the first to suggest that exposure to repeated HLB is associated with elevated risk of PTSD compared to single HLB. Specifically, compared with those with no HLB exposure, the relative odds of reporting PTSD nearly doubled from 4.85 to 8.55 for those with single vs. repeated HLB, respectively. Furthermore, the current research extends previous findings with animals and archival medical records suggesting that occupational risk of LLB was also associated with significantly increased risk of PTSD (1, 40, 43–45). The significant interaction of HLB and LLB herein suggests that those with occupational risk of LLB may be at elevated risk for diagnoses of PTSD compared to their lower risk counterparts even in the absence of an HLB, but is more pronounced following HLB exposure. Taken together, these findings add to the growing body of evidence that overpressure exposure is associated with greater risk of PTSD, though the mechanism for this effect (e.g., physiological damage to the brain, psychological trauma, an inflammatory mechanism) has yet to be elucidated.

Whereas, previous findings with hearing loss, tinnitus, headaches/migraines, and PTSD have been clearly associated with overpressure exposure, only limited prior research has provided evidence of associations between overpressure and fatigue or depression (1, 11, 12, 46). Although prior research with animals documented an associated between overpressure and indicators of depression-like behaviors, there is not yet evidence of this pattern among humans (1, 11, 12). The current research suggests a similar pattern in humans, which may also inform our understanding of the possible associations between overpressure and suicide that has been posited elsewhere. Furthermore, this report is the first to document an association between overpressure exposure and self-reported diagnoses of chronic fatigue syndrome specifically, though again the mechanism for this effect is still unclear. For example, it is possible that subclinical symptoms arising from overpressure exposure may impair one's ability to get high-quality sleep. Alternatively, it is possible that the shockwaves associated with overpressure may damage certain regions of the brain like the thalamus, which has been implicated in sleep-wake cycles (47, 48). Given the importance of both suicide and sleep in military populations, understanding these associations more fully represents exciting avenues for future research (49–55).

### 4.1. Limitations

There are notable limitations that warrant mention. First, the measurement of blast exposure (both LLB and HLB) may have resulted in misclassification. For example, we used occupational risk as a proxy for repetitive exposure to LLB. However, merely working in one of these occupations at some point in one's military career does not necessarily equate to having sustained repeated exposure to LLB, nor does it allow for precise considerations of factors that might affect the relationship between such exposure and adverse health outcomes including proximity to the weapon system, type of weapon system, frequency or duration of exposure, personal protective equipment, etc. Additionally, the wording of the HLB exposure item also included exposure to bullets. It is also possible that some warfighters may have been referring to LLB exposure when self-reporting exposure to “blast/explosions.” Furthermore, the number of HLB exposures was forced to range between 0 and 99 due to survey design. Although these data were heavily skewed, it is not presumed to affect the results as logistic regression does not assume normality of predictors nor residuals (56, 57).

Second, there are several limitations associated with the use of self-reported physician diagnoses. Because of the project's exploratory nature, we examined all 45 available diagnoses and did not correct for multiple comparisons. While certain statistically significant effect estimates may be false-positive findings, we felt that this was appropriate due to the exploratory nature of the analysis. Additionally, the use of newly self-reported physician diagnoses may lead to censoring of some outcomes of interest, especially since HLB and LLB exposure was assessed on the 2011–2013 survey while participants in the first three enrollment panels had the opportunity to report on most outcomes of interest in previous surveys. The absence of validated dates of exposure limited our ability to precisely identify medical conditions that were truly new-onset. Our decision to use newly reported diagnoses (thereby excluding those who had previously reported the diagnosis) was motivated by the need to exclude prevalent conditions, but may result in underestimates of the association between blast exposure and diagnoses. Five of the conditions examined (i.e., high cholesterol requiring medication, kidney stones, acid reflux/gastroesophageal reflux disease requiring medication, tinnitus/ringing of the ears, and impaired fecundity) were added to the survey in 2011 and were thus assumed to be new-onset even though the conditions may have emerged earlier. Additionally, the present research used self-reported diagnoses by a physician rather than official medical records of diagnosis. Because we do not have precise measures of the date of blast exposure, utilization of self-reported diagnoses afforded more efficiency in examining new-onset of these medical conditions as precise dates of exposure would be necessary to identify which diagnoses onset after the exposure. Although there are several limitations associated with self-report, self-reported diagnoses in the Millennium Cohort appear to have adequate agreement with official medical records (58). Additionally, by relying on self-reported rather than official diagnoses incorporated in the medical record, the present study was able to examine outcomes among those still currently serving in the military and veterans regardless of their utilization of the VA healthcare system or other insurance coverage, which is a notable addition to previous literature.

### 4.2. Future work

Despite these limitations, the current research suggests that there may be adverse health outcomes associated with overpressure exposure, including single HLB exposure, repeated HLB exposure, and repetitive LLB exposure. Although the current research articulates the association between these exposures and self-reported clinical diagnoses, it would also be worthwhile to examine whether service members and veterans also report subclinical symptoms, which may drive healthcare-seeking behavior. Additionally, future research can further investigate the medical conditions noted herein using more precise measures of overpressure exposure (e.g., impulse overpressure, distance from the blast, presence of personal protective equipment). Furthermore, additional research on the long-term implications associated with both single and repeated TBIs caused by both blast exposure and direct impact to the head are warranted.

### 4.3. Conclusions

Taken together, the findings herein suggest that overpressure exposure increases the likelihood of several self-reported diagnoses including PTSD, hearing loss, chronic fatigue syndrome, tinnitus, neuropathy-caused reduced sensation in the hands and feet, depression, vision loss, sinusitis, reflux, and anemia. Furthermore, the data reported herein provide additional support for the idea that the combination of HLB and LLB exposure may be associated with greater risk of migraines, PTSD, and impaired fecundity, and may adversely affect performance. These findings provide further evidence of the potential adverse consequences associated with overpressure exposure and underscore the necessity of public health surveillance initiatives for blast exposure and/or safety recommendations for training and operational environments.

## Data availability statement

The datasets presented in this article are not readily available because of institutional regulations protecting service member survey responses, but may be available from the corresponding author on reasonable request following receipt of appropriate regulatory approvals. Requests to access the datasets should be directed to Millennium Cohort Study Principal Investigator, DoD.MillenniumCohortPI@health.mil.

## Ethics statement

The study protocol was approved by the Naval Health Research Center Institutional Review Board in compliance with all applicable Federal regulations governing the protection of human subjects. Research data were derived from an approved Naval Health Research Center Institutional Review Board protocol, number NHRC.2000.0007. The patients/participants provided their written informed consent to participate in this study.

## Author contributions

JB, RR, and DT contributed to the conception of the study. JB, CK, RR, and DT contributed to the design of the study. JB and CK prepared the data for analysis. JB performed the statistical analyses and wrote the first draft of the manuscript. All authors contributed to manuscript revision, read, and approved the submitted version.
